# Feasibility of a method for low contrast CT image quality assessment using difference detail curves for abdominal scans

**DOI:** 10.1016/j.zemedi.2022.01.001

**Published:** 2022-02-17

**Authors:** Christian Sommer, Ismail Özden, Mathias S. Weyland, Carolina Duran, Gerd Lutters, Stephan Scheidegger

**Affiliations:** 1 ZHAW School of Engineering, 8401 Winterthur, Switzerland; 2 Fachstelle Strahlenschutz, Kantonsspital Aarau, 5000 Aarau, Switzerland

**Keywords:** Low contrast CT, CT image quality, Difference detail curve, CT phantom

## Abstract

This work describes a measurement method for assessing dose-related image-quality of CT scans based on the difference detail curve (DDC) method, and showcases its use in a low contrast setting. The method is based on a phantom consisting of elliptical slices of different sizes into which contrast object modules can be inserted. These modules contain contrast objects based on (synthetic) resin mixtures with sucrose (native) or sodium iodine (contrast medium). Mixing ratios are provided to achieve a range of clinically relevant CT-numbers with these materials. The phantom is characterized in terms of contrast accuracy, energy dependency and long-term drift with satisfying results. Contrast accuracy and energy dependency are similar to that of water or soft tissue. Image quality of 655 scans of the phantom acquired at 30 different clinical institutions and with 16 different CT scanner models from 4 manufacturers was assessed by calculating a difference detail curve (DDC) from evaluation of up to 5 human observers using a custom-made software (RadiVates) described in this work. Based on these measurements, inter-observer variability was quantified using a bootstrap method and was shown to be a large contributor to the overall variability. This work demonstrates that assessment of CT image quality is feasible with the aforementioned phantom and DDC method.

## 1 Introduction

The desire for dose- and image quality co-optimization in Computer Tomography (CT) scans [1] presumes the ability to measure radiation exposure and image quality as realistically as possible, in phantoms or preferably in patients. While the quantification of dose is readily addressed by measuring the dose length product (DLP), assessments of image quality are far more challenging [2]. In particular, image quality in this context refers to image quality as seen by the radiologist while performing clinical tasks. In this realm of CT image quality, object detectability is a pivotal quantity, especially when iterative reconstruction algorithms are involved: Since these algorithms behave in a non-linear way, the total modulation transfer function (MTF) does not correspond to the product of the partial MTFs and thus the MTF is an inadequate figure of merit for image quality. However, low contrast detectability is in general an important point of reference for clinical use.

In order to achieve the goal of dose- and image-quality co-optimization, we propose a phantom and characterize it regarding to its contrast accuracy, stability of the materials used and dependency on the X-ray spectrum employed. A precursor phantom was developed in 2017 [3]. In that phantom, the contrast objects were grouped by contrast and aligned along signal-to-noise-ratio (SNR) isolines. In-house testing with this phantom revealed that observers were able to recognize these patterns and infer the presence of unperceived contrast objects. This weakness holds similarly for the CATPHAN phantom (The Phantom Laboratory, Greenwich, NY). In addition, Gulliksrud et al. [4] found that the CATPHAN suffers from inter-phantom variations in the low contrast resolution, and Annkah et al. demonstrated that the CATPHAN does not represent the tissue in the human body in an accurate form [5]. To overcome these shortcomings, we developed a modular phantom for simultaneous DLP measurement and assessment of low-dose object detectability.

For assessing low-dose object detectability, we propose and showcase an approach with the aforementioned phantom. The approach is based on a difference detail curve (DDC) [3] calculated from data generated by human observers. The DDC method is an indicator for the minimal contrast of objects perceived, as a function of their sizes. In principle, the shape of the curve provides information about the low contrast detectability as well as the high contrast detectability [6]. Unlike the contrast detail curve, the DDC is more robust against misinterpretations which may occur from CT related variables [7]. Since the DDC is calculated from data provided by human observers, special interest is paid to how the resulting DDCs differ between these observers (inter-observer variability).

## 2 Materials and methods

In the following sections, we describe the geometry of our phantom (Section 2.1), the material composition of the contrast objects used in our phantom (Section 2.2), the methods and software used for evaluations of the CT scans of our phantom (Section 2.3), and the setup, scan protocols and parameters used for validation and measurements (Section 2.4).

### 2.1 Body phantom geometry

**Figure 1:**
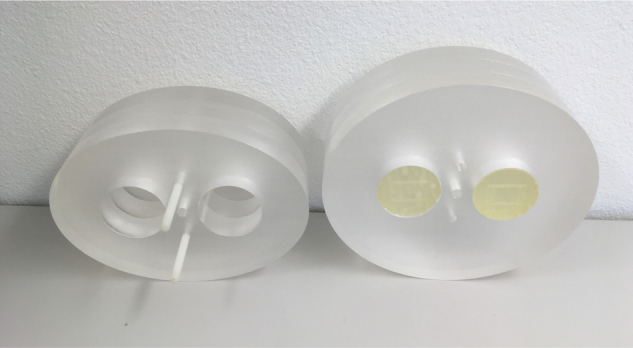
Two body phantoms of different size. The left phantom has an effective diameter of 27 cm and the right one an effective diameter of 32 cm. Two DDC modules are installed in the right phantom, the corresponding holes in the left phantom are unpopulated.

The proposed body phantom is designed for testing abdominal CT scans. It consists of elliptical polymethyl methacrylate (PMMA, casted – clear) slices in five different effective diameters (16 × 22, 20 × 24, 24 × 32, 28 × 34 and 30 × 34 cm^2^) of 4 cm depth each, which can be stacked using two rods of polyamide (PA6.6). Each slice has two holes for these rods, a central hole for dose measurements and two 80 mm holes where pluggable inserts (DDC modules, see below) can be placed. If no module is used, the hole is plugged with a dummy PMMA insert during measurements. The benefit of this modular approach is that different modules can be used and furthermore be modified independently of the PMMA body. All results shown in this work were made with three 24 × 32 cm^2^ slices (body phantom with 27 cm effective diameter) or three 30 × 34 cm^2^ slices (body phantom with 32 cm effective diameter). According to the American Association of Physicists in Medicine (AAPM) size specific dose estimation (SSDE) report [8], an effective diameter of 27 cm refers to a patient age of 16 while a phantom size of 32 cm effective diameter is comparable to the Standard IEC CTDI Phantom [9], motivating the aforementioned choice. These configurations are shown in Figure 1 without the additional slices normally added for the reduction of edge artefacts and used as scatter material.

**Figure 2:**
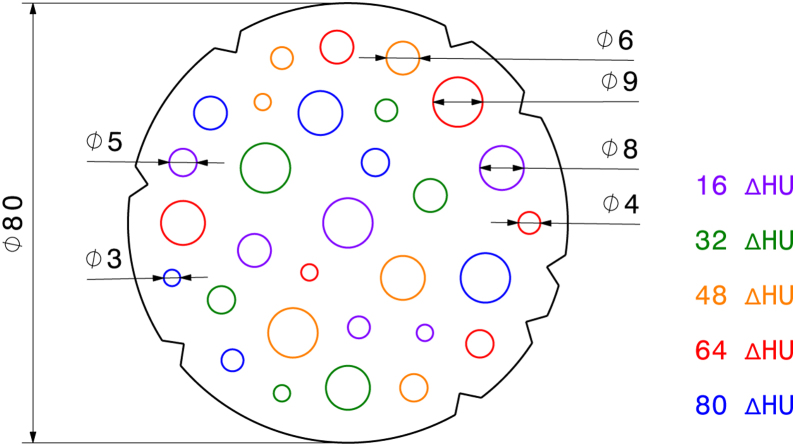
Drawing of a 80 mm DDC module for insertion into the body phantom. Each colour corresponds to a different contrast value; the contrast objects are positioned randomly. The triangular cutouts on the outside are for automatic identification and position detection.

The DDC modules are cylindrical structures with embedded contrast objects, from which difference detail curves (DDC) [3] are finally calculated. Two types of DDC modules were constructed: One for contrast-optimized protocols (no. 1) and one for the use with native protocols (no. 2). Each module consists of 30 randomly positioned cylindrical contrast objects (Figure 2), and the modules are placed far away from the phantom periphery to enforce contrast object positions in an area of similar SNR.

**Figure 3:**
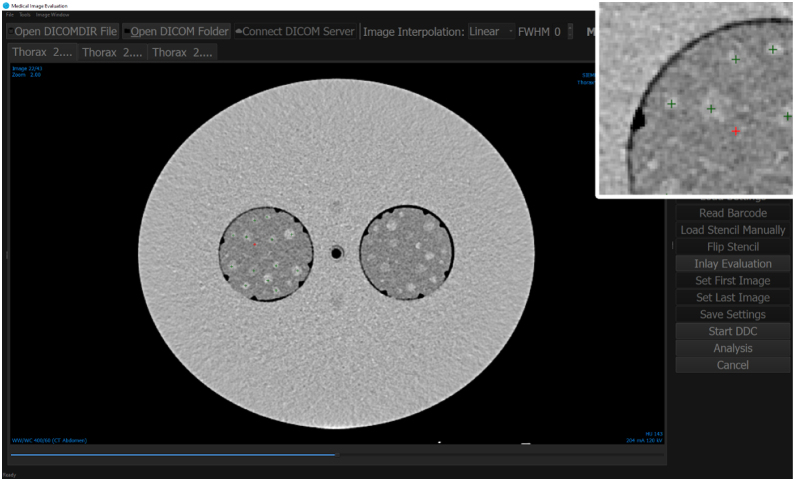
Analysis of the DDC modules from an CT scan with both modules from an Siemens Somatom Definition AS with a chest scan protocol, 120 kV tube voltage and 294 mA tube current. The module with contrast agent (no. 1) is on the left side while the native DDC module (no. 2) is on the right side. An abdomen window (ww:400 and wc:60) is used. The figure shows our software “RadiVates”. The overlay at the top right was magnified by 300%. The green crosses are contrast objects which were seen by the observer while the red cross designates a false click. The software runs in dark mode during the DDC evaluation.

The contrast objects are rods of six different diameters (3–9 mm) made from epoxy resin mixtures with five different contrast values (Figure 2). Details on the rod materials and casting are given in Section 2.4. Each module has a depth of 40 + 0/−2 mm and therefore fits in a single slice of the phantom body. For automatic identification and position detection, eight triangular cutouts are milled into the barrel, and a unique barcode is milled into the rod's face. Figure 3 shows CT scans of the phantom with DDC modules inserted.

### 2.2 Material composition of contrast object

The base material for the modules is clear epoxy (Translux D150, Axson Technologies, Chassieu, France). A 100:60 ratio of resin to hardener was used in a compromise between hardness and the desire to obtain a baseline CT number of 0 Hounsfield Units (HU). For the module with contrast agent (no. 1), the resin was mixed with a solution of sodium iodine (NaI) in distilled water. For the native contrast module (no. 2), sucrose was admixed. The target contrasts were chosen in the range from 16 ΔHU to 80 ΔHU. Starting from calculations based on material density, the ratios and weight proportions to obtain these target contrasts were iteratively refined and are reported in Table 1 as well as in [3]. The contrast of the materials is required to be stable over time as well as to closely match the targeted contrast.

**Table 1:** Material composition by per cent per weight for each target contrast (ΔHU) in the two modules. The solution for the module with contrast agent is a 1:1 mixture of sodium iodine (NaI) powder and distilled water.

ΔHU	Contrast agent		Native	
	Translux D150 (%)	NaI solution (%)	Translux D150 (%)	Sucrose (%)
16	99.82	0.18	95.60	4.40
32	99.60	0.40	91.11	8.89
48	99.39	0.61	86.60	13.40
64	99.17	0.83	82.10	17.90
80	98.96	1.04	77.61	22.39

The rods were cast into a negative mould made from silicone, which was mounted in a curing assembly. To prevent segregation of resin and hardener, this assembly was under constant rotation (10 rpm) while curing. Because of possible air inclusions, air ducts were designed into the faces of the silicone negative and the assembly. Furthermore, the resin mix was degassed in a vacuum chamber prior to casting. In a second step, the rods were cast in epoxy to obtain the final module. Figure 3 shows a CT scan of both modules inserted into the body phantom.

### 2.3 DDC evaluation and RadiVates software

To investigate the feasibility of the use of difference detail curves (DDCs) for assessing image quality of the CT scans mentioned in Section 2.4, DDC plots were generated according to the following procedure: On the horizontal axis denoting object size, the different diameters *s* of contrast objects are reported (in mm). The vertical axis denotes the lowest contrast Δ_min_*HU* of that size still seen by observers. Visual inspection reveals a logarithmic relationship between these two variables. In general, a logarithmic relationship between the magnitude of a stimulus and its perceived intensity are very common and well documented in the field of psychophysics (e.g. Weber's law, Fechner's law, Fitts's law, Hick's law) and may ultimately root in the Shannon–Hartley theorem of information theory [10].

For the expected value E of Δ_min_*HU*, the model

(1) $$E\left[\Delta_{\min}\mathrm{HU}(s)\right]=\alpha +\beta \log\left(s\right)$$

with parameters *α* and *β* follows from the aforementioned logarithmic laws. Because DDCs are evaluated by different observers which may introduce an observer-bias, it is critical to quantify this bias and to assess the robustness of the parameter estimates in the light of inter-observer variability. In order to do so, the model is extended with observer-specific parameters $\alpha_{i}^{\prime }$ and $\beta_{i}^{\prime }$ for observer *i*:

(2) $$E\left[\Delta_{\min}\mathrm{HU}(s)\right]=(\alpha +\alpha_{i}^{\prime })+(\beta +\beta_{i}^{\prime })\log\left(s\right)$$

Since this model is underspecified for least-square optimization, an additional constraint has to be introduced. A natural choice is $\sum_{i}\alpha_{i}^{\prime }=\sum_{i}\beta_{i}^{\prime }=0$, i.e. the average effect is given by *α* and *β*, respectively, and the observer-biases are 0 on average.

Robustness is assessed using a bootstrap resampling technique [11]: For a selected scan evaluated by *n* observers, *m* least square fits are performed on a randomly sampled subset of DDCs of sample size *k* ≤ *n*. Hence, *m* parameter estimates *θ*_*kj*_ are obtained for each sample size *k* (1 ≤ *j* ≤ *m*). According to the bootstrap theory, the standard deviation of those parameter estimates yields their respective standard errors, i.e.

(3) $${\hat{\sigma }}_{\bar{\theta_{k}}}=\sqrt{\frac{1}{m-1}\sum_{j=1}^{m}{\left(\theta_{\mathrm{kj}}-{\bar{\theta }}_{k}\right)}^{2}}$$

A choice of *m* = 10^5^ was sufficient to obtain standard error estimates that were stable at one significant digit.

The evaluation of the DDC was done with up to five human observers under controlled light conditions and a medical grade display. A python software called “RadiVates” was developed for the evaluation by human observers (Figure 3). During evaluation, zoom and window parameters were fixed (a abdominal window with ww:400 and wc:60) and the software interface switched to a dark mode to minimize the effect of bright stimuli. The observers’ task was to click any contrast object he or she could clearly identify. Observers were not provided with any information on their performance (correctly identified and missed objects) throughout the process. Based on the clicks provided by an observer, the software found Δ_min_*HU*(*s*), from which the model parameters are calculated as discussed above.

### 2.4 Setup, scan protocols and parameter for verification and measurements

*Verification:* The targeted contrast as well as stability over time and energy dependency of the DDC modules were verified. These verification scans were made with DDC modules inserted into the phantom with effective diameter of 32 cm on a Siemens Somatom Definition AS. To measure stability over time and evaluate potential drift of the measurements, these scans were repeated over the course of 1.5 years. All measurements were done with an chest scan protocol and a reconstruction kernel “I30f” as well as a slice thickness of 2 mm. To compare this iterative reconstruction algorithm to filtered back projection, a second kernel “B30f” was used. In order to evaluate energy dependency of the contrast material, measurements were carried out with several tube voltages (80, 100, 120, 140 kV). Maximal tube current was selected to minimize image noise. To obtain the same tube current and tube voltage over all measurements, any dose modulation (e.g. automatic exposure control or care kV from Siemens) were disabled. The setup yields a CTDI_Vol_ of 11, 23, 40, 52 mGy at 80, 100, 120, 140 kV, respectively. The pitch was fixed to 0.6 and the slice diameter to 2.0 mm. The evaluation of these measurements was done with the RadiVates software (see Section 2.3). The HU values of all contrast objects and the surrounding area (epoxy) were quantified automatically with the help of a module template that identified these regions of interest (ROIs). The periphery of the ROIs were discarded to minimize the effect of inaccuracies in the template positioning. To reduce the ROIs a morphological transformation – erosion with a circle with 5 pixel diameter – was used. With this reduction, all contrast objects were still much larger than the resolution of the detector. Based on this, no partial volume effects are expected. Finally the mean, standard deviation, minimum, maximum and median values across all selected slices were extracted for each target HU value and each diameter as well as for the surrounding base material.

*Measurements with clinically used scan protocols:* 655 scans in total were made with protocols used for clinical diagnoses and evaluated by human observers. Scans were done with the body phantom with an effective diameter of 32 cm (30 × 34 cm) and repeated with the phantom with effective diameter of 27 cm (24 × 32 cm) to evaluate size-specific behaviour of the DDC method (see Section 2.3) as well as to cater for different patient body sizes. Different CT scanner models in different institutions were used (see Table 2), employing clinical scan protocols with fixed CTDI_Vol_ to probe a set of dose levels below and above the standard clinical protocol dose. The tube voltage was set to 100 or 120 kV, and tube current was chosen such that the CTDI_Vol_ calculated by the machine was on a level from 3 to 20 mGy, thus covering the clinically relevant range. Specifically, values of 3, 5, 7, 9 and 13 mGy (27 cm phantom), and 7, 9, 11, 13, 15, 20 mGy (32 cm phantom) were targeted.

**Table 2:** Summary of CT scanners used. The “# Institutions” column refers to the number of institutions at which the indicated device was used, and the “# DDCs” column refers to the total number of DDCs (see Section 2.3) that were obtained for that device.

Manufacturer	Device	# Institutions	# DDCs
Siemens	Definition AS+ (new Detector)	6	541
	Definition AS (new Detector)	4	244
	Definition AS (old Detector)	3	207
	Definition Edge	3	149
	Definition Flash (new Detector)	2	156
	Definition Flash (old Detector)	3	242
	Force	1	44
	Sensation 16	1	126
Toshiba	Aquilion	1	44
Philips	Brilliance 16	1	39
	Brilliance 64	1	36
	iCT 256	2	84
	Ingenuity Core	1	78
GE	Optima CT660	2	88
	Revolution EVO	2	76
	Revolution HD	1	36

## 3 Results and discussion

### 3.1 Verification

**Figure 4:**
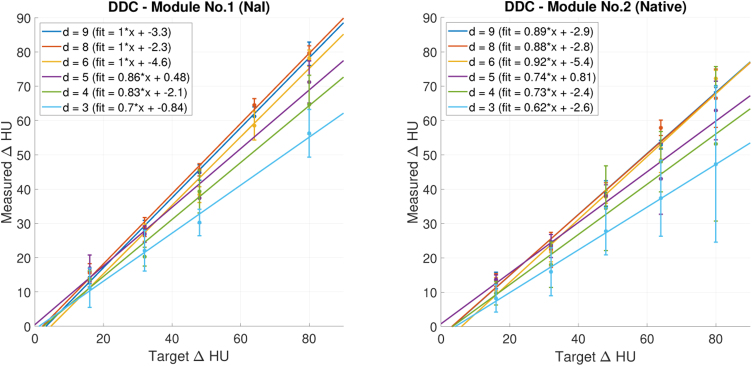
Dots: Measured contrast (ΔHU) for each diameter. The left figure shows the measurements with the module with contrast media (no. 1), while the right one is with the native module (no. 2). All measurements were done with 120 kV tube voltage and a chest scan protocol. Lines: Corresponding linear fits.

Figure 4 shows repeated phantom measurements with 120 kV tube voltage and a chest scan protocol with an “I30f3” kernel. Each diameter corresponds to a colour for which a linear regression line is calculated. The target contrast (Δ*HU*) is represented on the horizontal axis, the vertical axis shows the measured contrast (Δ*HU*). In a perfectly manufactured and reconstructed module, measured and targeted contrast would be equal and yield a regression line with a slope of 1 and an intercept of 0. While the linear regression represents the data well in general (each *R*^2^ > 0.97), a number of deviations from the aforementioned perfect case are observed: In the module with NaI contrast agent, the slope of the series with smaller diameters (3, 4 and 5 mm) is lower than expected and progressively decreases with the diameter. In the native module, the slopes are generally below 1, which could be addressed by adapting the ratios given in Table 1.

**Figure 5:**
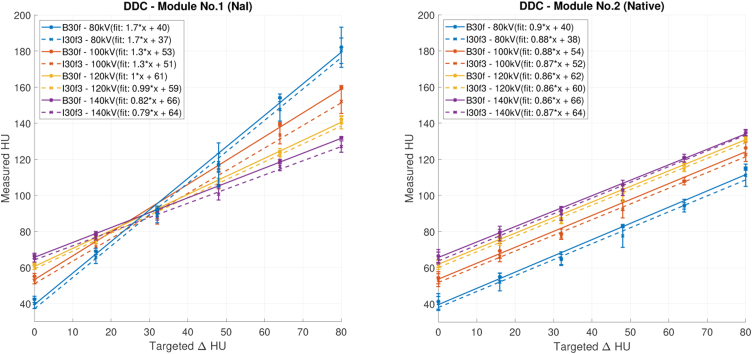
Effect of different tube voltages on measured Hounsfield Units (HU). The left figure shows the measurements with the contrast agent module (no. 1) and the right figure those with the native module (no. 2). The solid line represents measurements with a “B30f” kernel and the dashed lines thus with a iterative “I30f3” kernel. A chest scan protocol with 2 mm slice thickness was used on a 9 mm contrast object.

In Figure 5 the impact of tube voltage and kernel on scans with a chest scan protocol are shown. The solid lines denote the measurements with a “B30f” kernel and the dashed line are the same measurements with a “I30f3” kernel involving iterative reconstruction. The contrast objects with 9 mm diameter were evaluated because results were expected to be most reliable with the largest structures. The surrounding base material (epoxy) of the module is shown as 0 ΔHU targeted. The measurements with the module with contrast agent (no. 1) are shown on the left, while the native module (no. 2) is on the right.

For both modules, the curves describing the targeted contrast in Figure 5 have the same intersect with abscissa since the used material is similar to the surrounding base material (Translux D150). While the slopes of the results with the native contrast module all are equal, the slopes from the module with contrast agent depend on the tube voltage. This is expected, since contrast agents have a higher impact on the represented HU than materials with low atomic number *Z* [12]. The base material is approximately 60 HU and as the targeted tissue equivalent material but corresponds to blood and liver [13].

**Figure 6:**
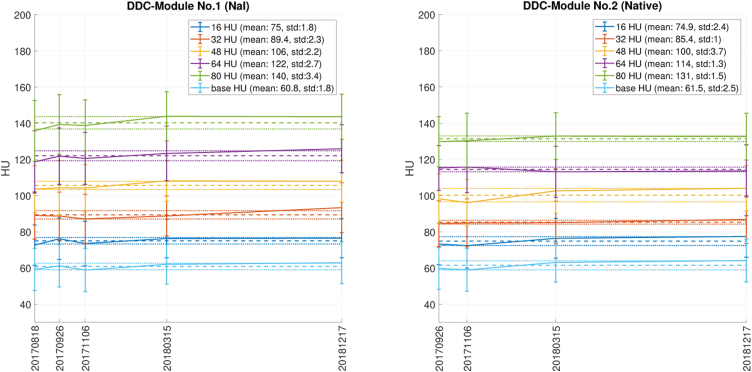
Drift of contrast with a chest scan protocol with kernel “I30f3” and 120 kV tube voltage. The left plot shows the module with contrast agent (no. 1) and the plot on the right shows the native module (no. 2). Colours are assigned according to contrast series and surrounding base material (light blue). The solid lines show the mean of the contrast objects with 9 mm diameter, with standard deviation as error bars. The dashed line is the mean of all measurements shown while the dotted line indicate the standard deviations of these means. The horizontal axis shows the date when the measurement was acquired while the vertical axis shows the measured contrast in Hounsfield Units (HUs).

Figure 6 shows the measured drift in time with a tube voltage of 120 kV and a chest scan protocol with an “I30f3” kernel. As in Figure 4, 9 mm contrast objects were considered since the most reliable measurements were expected in the largest structures. The light blue line shows the surrounding base material (epoxy) while the other colours represent the different contrast series. During this time span each module (nos. 1 and 2) received an accumulated dose of 336 Gy. Considering potential contrast drift in time, no relevant change was observed. Thus there is no indication of material diffusion.

### 3.2 Inter-observer variability and robustness

As mentioned in the introduction, inter-observer variability is a crucial aspect of any image quality assessment that is based on human observer data. In order to investigate this aspect with respect to the DDC method, the subset of DDCs is selected subject to the following constraints: All DDCs in the subset have been acquired from CT scans at the same institution, with the same CT scanner and with the same tube voltages and CTDIs, and the scans were evaluated by all 5 observers. Some observers may provide more than one DDC in the sense of a repeated measure. The fact that such curves are not statistically independent is correctly reflected in the parameter estimates obtained by minimizing the sum of square error of Eq. (2), but not in the subsequent bootstrap. The resulting parameter estimates ($\hat{\alpha }$ and $\hat{\beta }$) are shown in Table 3 along with the corresponding standard errors (${\hat{\sigma }}_{\bar{\alpha }}$ and ${\hat{\sigma }}_{\bar{\beta }}$) and the standard deviations of the observer-bias (${\hat{\sigma }}_{\alpha^{\prime }}$ and ${\hat{\sigma }}_{\beta^{\prime }}$).

**Table 3:** Parameter estimates for a DDC set of 5 curves obtained from CT scans acquired at 120 kV with a CTDI of 9 mGy. *θ* ∈ {*α*, *β*}.

Parameter (*θ*)	Estimate ($\hat{\theta }$)	Standard error (${\hat{\sigma }}_{\bar{\theta }}$)	Std. deviation of observer-bias (${\hat{\sigma }}_{\theta^{\prime }}$)
*α*	115.71	16.056	27.48
*β*	−32.21	7.245	9.90

Estimates for the parameters $\alpha_{i}^{\prime }$ and $\beta_{i}^{\prime }$, i.e. the observer-specific parameters, are reported in Table 4 with constraints of $\sum_{i}\alpha_{i}^{\prime }=0$ and $\sum_{i}\beta_{i}^{\prime }=0$. In both cases, the observer-specific parameters in Table 4 are roughly one order of magnitude smaller than the overall parameters in Table 3.

**Table 4:** Minimal contrasts (HU) seen by 5 observers. “–” indicates that an observer did not see any contrast object of that size. $\alpha_{i}^{\prime }$ and $\beta_{i}^{\prime }$ are observer-specific parameters for observer *i*.

Observer *i*	Diameters						Estimates	
	3 mm	4 mm	5 mm	6 mm	8 mm	9 mm	$\alpha_{i}^{\prime }$	$\beta_{i}^{\prime }$
Observer 1	64	48	32	32	32	48	−41.275155	14.8745202
Observer 2	–	80	80	80	48	48	24.742225	−8.9157413
Observer 3	–	80	64	80	48	48	16.209390	−5.8408181
Observer 4	64	80	80	48	48	48	−1.489175	0.5371008
Observer 5	–	80	64	48	48	48	1.813253	−0.6529743

**Figure 7:**
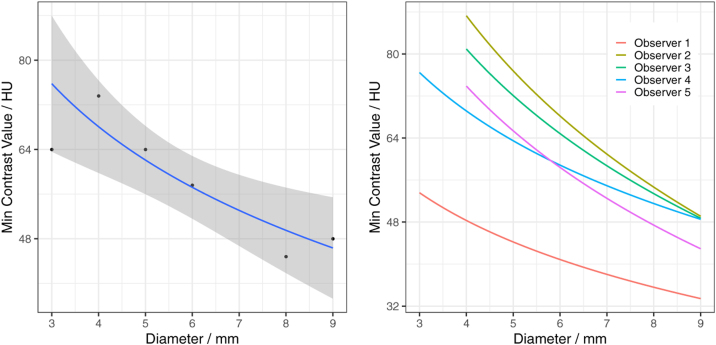
Aggregated (left) and individual (right) DDCs from the dataset described in Section 3.2 and reported in Table 4.

The resulting DDC predictions are shown in Figure 7, aggregated for all observers on the left and with an individual fit for each observer on the right. The regression lines are similar for all observers except observer 1, which outperforms the other observers. This is also reflected in a low $\alpha_{1}^{\prime }$ (see Table 4).

Following the bootstrap-approach outlined in Section 2.3, the standard errors of the two parameters are reported in Table 5. As expected, the error increases as the number of observers, *i* is reduced. In a realistic clinical setting, the number of observers is very limited, thus there is interest to keep *i* low. Providing a suggestion for a particular *i* is challenging since it depends on maintaining an error boundary that is chosen arbitrarily. For a quantitative approach, however, we propose to compare the standard error of a parameter ${\hat{\sigma }}_{\bar{\theta }}$, as given in Table 3, to the one given in Table 5.

**Table 5:** Standard errors of parameter estimates according to the bootstrap procedure described in Section 2.3. *k* is the number of DDCs randomly selected for each iteration.

Standard error	*k* = 5	*k* = 4	*k* = 3	*k* = 2	*k* = 1
${\hat{\sigma }}_{\bar{\alpha }}$	14.10436	16.24794	19.08222	23.13647	29.93254
${\hat{\sigma }}_{\bar{\beta }}$	6.155096	7.106872	8.373405	10.22386	13.45809

### 3.3 Evaluation of scan protocols for clinical diagnosis

**Figure 8:**
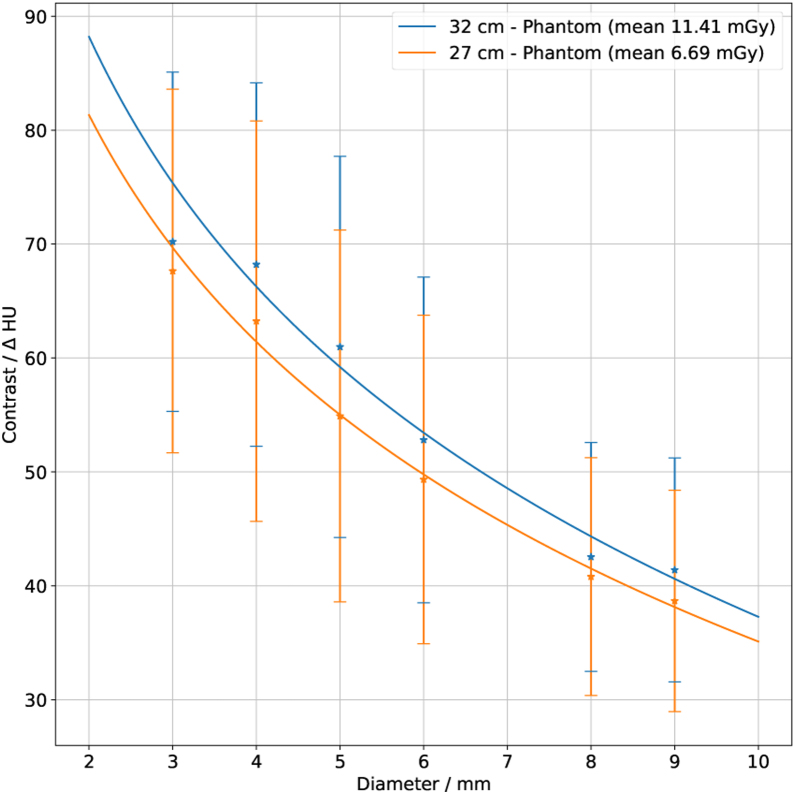
Aggregation of all DDCs for each of the two phantoms. The blue curve shows the results from the large body phantom (32 cm effective diameter) while the orange curve shows the results from the small body phantom (27 cm effective diameter) and both modules. A fit according to Eq. (1) is applied; the error bars show the standard deviations.

All DDCs obtained from the two body phantoms with measurements from protocols for clinical diagnosis are shown in Figure 8. The plot shows a fit over all observers, tube voltages, CTDI values, and DDC modules for each of the two phantoms.

The evaluated DDC points correlate well with the applied fit, allowing for interpolation of values that were not measured. The large error bars are a consequence of the huge parameter-variability within the respective groups.

**Figure 9:**
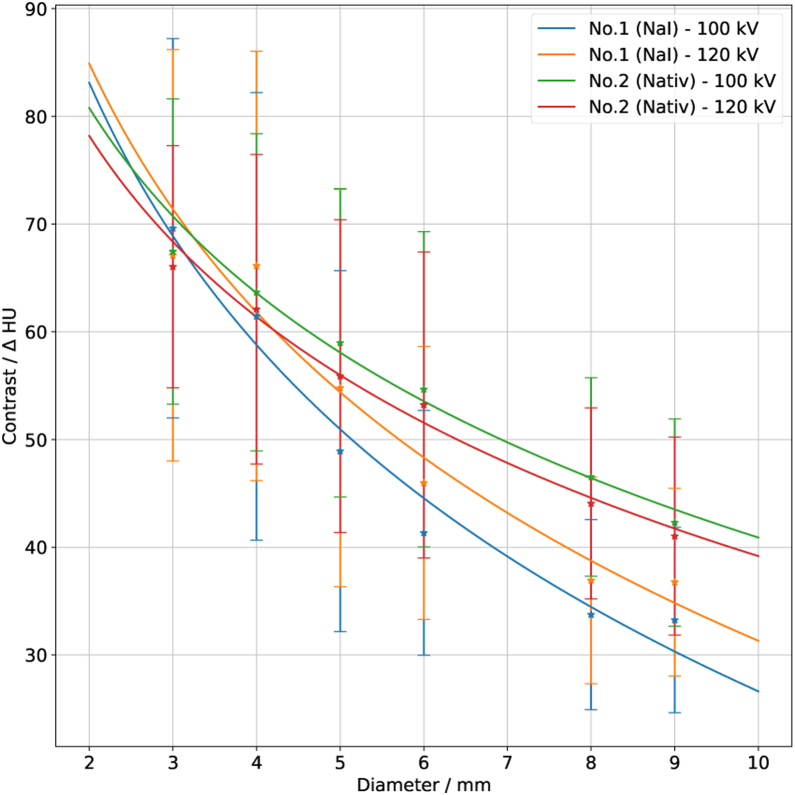
All DDCs from all observers with the body phantom with 27 cm effective diameter. Measurements which were done with the DDC module with contrast agent are shown by the blue curve for a tube voltage of 100 kV and by the orange curve with 120 kV. The results from the native DDC module are represented by the green curve (100 kV tube voltage) and red curve (120 kV tube voltage). The error bars show the standard deviation of all observers; these error bars are large since a large number of CT units and different CTDI values are included. A logarithmic fit was applied according to Eq. (1).

The difference between tube voltages and DDC module types for the body phantom with an effective diameter of 27 cm is shown in Figure 9. While the two curves for the native module almost overlap, those for the module with contrast agent do not, suggesting that the measured contrast is affected by the tube voltage.

A comparison between the phantom presented here and a precursor phantom with liquid low contrast objects [3] acquired at a similar dose level (mean CTDI = 10.87 mGy, images from 20 scanners were used), revealed in tendency a better visibility of the liquid-filled objects (*α* = 93.12 and *β* =−29.29, *R*^2^ = 0.9381 for the liquid-filled contrast objects versus *α* = 114.6 and *β* =−35.42, *R*^2^ = 0.9682 for the solid contrast objects presented in this study). This tendency is present in comparisons with a single observer as well as with groups of observers, but the difference is not significant. The DDC phantom presented by Sommer et al. [3] contains more contrast objects arranged along lines, whereas the phantom presented here has a smaller number of randomly arranged objects. The results support the hypothesis that the random-ordered objects are less detectable. However, the smaller number of contrast objects seems to be sufficient for DDC evaluation, since the DDCs of old and new phantoms exhibit a shift but do not differ fundamentally in shape.

## 4 Conclusions

The verification of the phantom revealed minor issues with the contrast values: In particular for smaller diameters, the measured contrast deviated from the targeted one. However, these measurements are well represented by the linear regression line, suggesting that a slight correction in the mixing rations would further improve the contrast accuracy of the modules. Considering a potential contrast drift in time, no relevant change was observed. The contrast of the module with contrast agent was affected by the tube voltage, while the native contrast objects, as well as the Translux epoxy, was only marginally affected. The Translux epoxy has a CT-number of around 60 HU at a tube voltage of 120 kV and is therefore closer to water or soft tissue than the PMMA material typically used for phantoms, but also has a lower density compared to, e.g. a standard CTDI phantom. While liquid phantoms [3] would naturally also provide CT-numbers close to water or soft tissue, the epoxy used in this phantom has clear advantages regarding ease of use and avoidance of air inclusions. Thus overall the used materials fulfill the requirements and are useful for that purpose.

The work demonstrates the use of DDC plots for co-assessing radiation dose and image quality. Inter-observer variability was shown to be substantial. Since DDCs are easier to compare when originating from the same set of observers, they should be acquired with this in mind when different protocols are to be compared. Furthermore, it is suggested to track observer performance, as it is likely that an observer may improve her/his skills over time. The logarithmic nature of the DDC was demonstrated and potential explanations were provided.

The measurements on different sites and evaluated by multiple human observers show that the DDC is a useful tool for protocol optimization regarding dose and image quality. With a library of measurements, a DDC reference catalogue (atlas) could be created which allows the comparison of different clinical protocols as well as different CT manufacturers, types and software revisions.

## Conflict of interest

None declared.
